# FKBP5 Regulates Osteogenesis of Human iPSC‐Derived Mesenchymal Stem Cells via FKBP5‐AKT‐FOXO1 Pathway

**DOI:** 10.1111/jcmm.70849

**Published:** 2025-10-31

**Authors:** Xiao‐Yu Tian, Biao Zhu, Xiang‐Bin Zhou, Wen‐Can Fang, Ye Lei, Meng‐Nan Liu, Ning Wu, Ning Wen, Hong Li

**Affiliations:** ^1^ Beijing Institute of Pharmacology and Toxicology Beijing China; ^2^ Department of Stomatology, The First Medical Center Chinese PLA General Hospital Beijing China; ^3^ Medical School of Chinese PLA Beijing China; ^4^ Department of Stomatology, Fuxing Hospital Capital Medical University Beijing China

**Keywords:** critical‐sized calvarial defect, FK506 binding protein 5, induced pluripotent stem cells, mesenchymal stem cells, osteogenesis

## Abstract

The induced pluripotent stem cells derived mesenchymal stem cells (iMSCs) have shown great promise for bone tissue regeneration in critical‐sized calvarial defects. Still, the roles of FKBP5 in its osteogenesis are rarely known. It was observed that FKBP5 increased rapidly in iMSCs following osteogenic differentiation. To elucidate its role, FKBP5 was knocked down or overexpressed by lentivirus infection. Interestingly, the down‐regulation of FKBP5 impaired the osteogenesis of iMSCs, whereas the up‐regulation of FKBP5 promoted it. Proteomics analysis of iMSCs/oeFKBP5 and iMSCs/oeNC revealed that the protein variances are enriched in several signalling pathways associated with osteogenesis. Notably, the PI3K‐AKT signalling pathway was enriched highly at both D4 and D14. Co‐immunoprecipitation results demonstrated that the binding proteins of FKBP5 are AKT and pS473‐AKT, but not PI3K/p‐PI3K or FOXO1/pS256‐FOXO1; however, the ratios of pS473‐AKT/AKT and pS256‐FOXO1/FOXO1 were down‐regulated by FKBP5. FOXO1 inhibitor AS1842367 lessened the enhanced osteogenesis by FKBP5. Moreover, in a rat critical‐sized calvarial defect model, the iMSCs/oeFKBP5 delivery exhibited improved bone regeneration capability than iMSCs/oeNC in vivo. In conclusion, our findings first revealed that FKBP5 promotes the osteogenic differentiation of iMSCs partially through the FKBP5‐AKT‐FOXO1 pathway and presents a promising approach to iMSCs transplantation for clinical bone defects.

## 1 Introduction

Supercritical large area or large segment bone defects, frequently brought about by trauma, tumour excision, inflammatory injury, and other factors, are clinical thorny issues. Exceeding the physiological regenerating capability, bone defects cannot be repaired by themselves and eventually may result in loss of bone function. Due to the limitations of the patient's physical condition and the vast volume of bone, the clinical application of autogenous bone grafting is extremely difficult to carry out.

Since being discovered firstly in bone marrow in 1976, mesenchymal stem cells (MSCs) have been found in various human body tissues, such as the umbilical cord, placenta, adipose tissue, dental pulp, etc. [1, 2]. Given that MSCs can differentiate into osteocytes, chondrocytes, and adipocytes, MSCs‐mediated therapy has proven safe and effective in the treatment of degenerative diseases and tissue defects [3, 4]. However, human tissue‐derived MSCs have limited proliferative capacity and undergo senescence after a few passages. Moreover, cell qualities vary significantly among donors because of age, body mass index, gender, and disease state, which makes their applications complicated [5, 6]. Therefore, it is crucial to have homogeneous and stable MSCs for effective clinical treatment.

In 2007, Lian et al. found that functional MSCs can be generated from human induced pluripotent stem cells (iPSCs), known as iMSCs [7]. Due to their comparable capacities for self‐renewal and differentiation to embryonic stem cells, iPSCs can be used to generate a large quantity of standardised and high‐quality iMSCs. Importantly, iMSCs derived from individual‐specific somatic cells possess the same genetic background, making them immune privileged for cell therapy in recipients [5]. These advantages have positioned iMSCs as an appealing option for obtaining a substantial population of stem cells for regenerative medical applications [8, 9, 10].

FK506 binding protein (FKBP) 5 is a 51‐kDa immunophilin that belongs to the family of FK506‐binding proteins, which are named after their capability to bind the immune suppressant FK506. FKBP5 consists of an FKBP‐type peptidyl‐prolyl cis‐trans isomerase domain (FK1), an FKBP‐like domain (FK2), and a three‐unit repeat of the tetratricopeptide repeat (TPR) domain, which can bind the MEEVD motif of other proteins [11, 12]. FKBP5 is a molecular chaperone, and one of the hallmarks of molecular chaperones is heat‐shock protein 90 [13, 14], as part of a heterocomplex that interacts with the members of the nuclear receptor family (glucocorticoid receptor, the mineralocorticoid receptor, oestrogen, and progesterone receptors, PPARg) [15, 16]. FKBP5 can also act as a scaffold protein to interact with many other cell signalling proteins (Akt, IKKα, IKKε and MAPK) [17, 18, 19], cytoskeleton proteins (TRAF2, Tau) [20, 21, 22], etc.

FKBP5 encoded by gene FKBP51, participates in physiological and pathological roles in regulating cell development, proliferation, survival, immune regulation, tumorigenesis, metabolism, and so on [23, 24, 25]. Previous studies also indicate that FKBP5 plays a crucial role in stem cell differentiation processes. FKBP5 promoted adipogenic differentiation of fibro‐adipogenic precursor cells [26]. Bta‐miR‐365‐3p is involved in the AMPK/mTOR signalling pathway in regulating Yanbian yellow cattle preadipocytes differentiation by targeting the FKBP51 gene [27]. FKBP5 controls cellular adipogenesis through p38 kinase‐mediated phosphorylation of GRα and PPARγ [28]. KLF15 positively regulated myoblast differentiation and muscle regeneration by activating FKBP5 expression [29].

Recently, Tang reported that FKBP5 promotes osteogenic differentiation of MSCs through type‐I interferon pathway inhibition [30]. A study in vitro reported that the expression of total and circular isoforms of FKBP5 was continuously increased during osteogenic differentiation. Notably, the top three up‐regulated circRNA in osteogenic differentiation are derived from FKBP51 in human bone marrow‐derived MSCs [31]. circ‐FKBP5 boosted BMSC proliferation and osteogenic differentiation by mediating the miR‐205‐5p/RUNX2 axis [32]. FKBP5 can serve as a biomarker for MSC osteogenesis [33]. In 2017, Lu B et al. first discovered that mutations in the FKBP51 gene can cause Paget's bone disease in the Chinese population [34]. However, the role and mechanism of FKBP5 in iMSC osteogenesis are unclear.

The objective of our study was to investigate the role and molecular basis of FKBP5 in the osteogenesis of iMSCs. To achieve this, lentiviral vectors were used to modulate FKBP5 expression, and then their impact and mechanism on osteogenesis potential in vitro were assessed. Furthermore, the in vivo effect of FKBP5 on osteogenesis was evaluated using a rat critical‐size calvarial defect model. Our findings may offer a novel approach for iMSCs transplantation in the clinical treatment of bone infarcts.

## 2 Materials and Methods

### 2.1 Cell Culture

iPSCs were purchased from ATCC Company (ACS‐1011, ATCC, USA). The cells were cultured in Nuwacell ncTarget culture medium (RP01020, Nuwacell, China). iMSCs were generated from iPSC using Nuwacell hPSC‐mesenchymal stem cell differentiation kit (RP01013, Nuwacell, China). iMSCs were cultured in Nuwacell ncMission culture medium (RP02010, Nuwacell, China) in a humidified atmosphere of 5% CO_2_ at 37°C. The cells were passaged every 4 days.

### 2.2 Cytometry Analysis

The iMSCs were collected and replaced in 1.5 mL EP tubes (1 × 10^4^ cells/tube). After rinsing twice with PBS, antibodies (Table S1) were added into each tube respectively and incubated on a flipping shaker at 4°C for 30 min. After washing twice with PBS, flow cytometry was used to identify the cells. The results were analysed with FlowJo software (BD, USA).

### 2.3 Osteogenic Differentiation and Staining

To induce osteogenic differentiation of iMSCs, the cells were seeded onto a gelatin‐coated plate and cultured in an osteogenic medium, which contained 50 μg/mL ascorbic acid (A4403, Sigma, USA), 100 nM dexamethasone (D4902, Sigma, USA), and 10 mM β‐glycerophosphate (G9422, Sigma, USA). To inhibit FOXO1 activity, AS1842856 (HY‐100596, MedChemExpress, USA) was used at a concentration of 1 μM.

After 7 days' induction, BCIP/NBT Alkaline Phosphatase Colour Development Kit (C3250S, Beyotime, China) was used for alkaline phosphatases (ALP) staining following the manufacturer's instructions. After 14 days' induction, the deposit of calcium salts can be depicted by Alizarin red staining (ARS) (ALIR‐10001, OriCell, China). The staining results were observed with light microscopy (DM IL LED, Leica Microsystems, Germany) and analysed with ImageJ software (National Institutes of Health software, USA) quantitatively.

### 2.4 Adipogenic and Chondrogenic Differentiation

The iMSCs were seeded at 1 × 10^4^ cells/cm^2^ for adipogenic differentiation. Adipogenesis differentiation media (RP02014‐A, Nuwacell, China) was used following the manufacturer's instructions. After induction for 7 days, adipogenesis was confirmed by staining with oil red O (OILR‐10001, OriCell, China). For chondrogenic differentiation, iMSCs counted to 4 × 10^5^ cells were harvested in a 15 mL conical tube. Human Stem Cells Chondrogenic Differentiation Kit (HUXXC‐90041, OriCell, China) was used according to the manufacturer's instructions. Alcian blue staining was performed post‐induction to confirm chondrogenesis at D21 after induction.

### 2.5 Lentivirus Infection

The FKBP5 shRNA lentivirus (HBLV‐h‐FKBP5shRNA‐ZsGreen‐PURO, shFKBP5) and corresponding knockdown negative control lentivirus (HBLV‐ZsGreen‐PURO negative control, shNC), FKBP5 overexpression lentivirus (HBLA‐h‐FKBP5‐3xflag‐ZsGreen‐PURO, oeFKBP5) and corresponding overexpression control lentivirus (HBLV‐ZsGreen‐PURO overexpression control, oeNC) were constructed (Hanbio Company, China). Three shRNA sequences for FKBP5 were designed, and their efficiencies for FKBP5 knockdown were tested in the HEK293 cells (Table S2). The knockdown efficiency of LV‐1039 was 75.8%; therefore, the shRNA sequence of LV‐1039 was selected for virus packaging of HBLV‐h‐FKBP5shRNA‐ZsGreen‐PURO. The shRNA sequence was 5′‐GGTATGAAATCGACTCCTTAA‐3′. Briefly, iMSCs were placed into plates at the density of 1 × 10^4^/cm^2^. The lentivirus was added to the medium (Multiplicity of infection, MOI = 30). The virus‐containing medium was then replaced by regular medium after 24 h. On D3 post‐infection, green fluorescence was observed under a microscope (DM IL LED, Leica Microsystems, Germany). Cells infected with knockdown or control lentivirus were grouped as iMSC/shFKBP5 and iMSC/shNC. Cells infected with overexpression or control lentivirus were grouped as iMSC/oeFKBP5 and iMSC/oeNC.

### 2.6 Real‐Time Quantitative Polymerase Chain Reaction (RT‐qPCR)

Total RNA was extracted using TRIzol (15596026, Sigma‐Aldrich, USA). RNA was converted to cDNA. Then target genes were amplified by PCR reagent according to the manufacturer's instructions (4993626, QIAGEN, Germany) with specific primers as described in Table S3. All samples were amplified in triplicate. The GAPDH served as an endogenous reference gene.

### 2.7 Western Blot

Cells were lysed in a lysis buffer (Beyotime, Shanghai, China). The samples of the same amount of protein were subjected to 10% SDS‐PAGE and then transferred onto PVDF membranes. After being blocked with 5% non‐fat dry milk at 37°C for 1 h, the membranes were probed with respective primary antibodies at 4°C overnight, and washed twice in TBS with 0.5% Tween 20 (TBST). Next, the membrane was incubated in a 1:5000 solution of HRP‐conjugated corresponding secondary antibody at room temperature for 1 h, then assayed by the enhanced chemiluminescence to display the strips. The antibodies used in the Western blot are listed in Table S4.

### 2.8 Comprehensive Proteomics Analysis of iMSC/oeFKBP5 and iMSC/oeNC

Cells counted to 2 × 10^6^ from each group (iMSC/oeFKBP5 and iMSC/oeNC) were collected, total protein was extracted from iMSC/oeFKBP5 and iMSC/oeNC (*n* = 3). The peptide mixture of each sample was labelled using TMT reagent according to the manufacturer's instructions (90104, Thermo Scientific, USA). The MS raw data for each sample were searched using the MASCOT engine 2.2 (Matrix Science, UK) embedded into Proteome Discoverer 1.4 software (Thermo Scientific, USA) for identification and quantitation analysis. The result was defined as significantly changed if *p* < 0.05 and fold change > 1.2. Cluster 3.0 (Michael Eisen, USA) and Java Treeview software (Java, USA) were used to perform hierarchical clustering analysis.

### 2.9 Co‐Immunoprecipitation (Co‐IP)

Classic Magnetic IP/Co‐IP Kit (88,804, Thermo Scientific, USA) was used for sample preparation. Briefly, iMSCs (1 × 10^7^) were lysed with IP Lysis/Wash Buffer, then the supernatant was collected to obtain whole cell protein samples (Input samples). Half of the supernatant was precleared with protein A/G agarose beads, then incubated with FKBP5 antibody‐coupled agarose beads overnight at 4°C. After several washes with IP Lysis/Wash Buffer, bound proteins were eluted by adding Laemmli buffer and incubating at 99°C for 10 min to obtain protein samples (IP samples). The primary antibody (Table S5), biotinylated protein ladder, horseradish peroxidase‐conjugated secondary antibody, and luminol‐peroxide were added to the separation module. Protein quantities were determined using a JESS digital Western blot instrument (ProteinSimple, USA) with a 12–230 kDa Separation Module (SM‐FL004, ProteinSimple, USA). Protein levels were analysed with Compass software (ProteinSimple, USA).

### 2.10 iMSCs Transplantation in the Rat Critical‐Sized Calvarial Defect Model

Animal experiments involved were approved by the Institutional Animal Care and Use Committee of the Beijing Institute of Pharmacology and Toxicology (Animal Ethics Approval No: IACUC‐DWZX‐2021‐666) and were conducted under the Guidelines for Animal Experimentation. Experiments of iMSCs transplantation are described below by ARRIVE guidelines. Male Sprague–Dawley rats (250–300 g) were purchased from SPF Biotechnology (Beijing, China).

The rats were randomly divided into four groups using a random number generator with a given range. Briefly, we employed a stratified randomisation method. First, rats were weighed and ranked based on body weight. According to this ranking, the 28 rats were stratified into 5 blocks based on weight: 250–260 g (*n* = 4), 260–270 g (*n* = 8), 270–280 g (*n* = 8), 280–290 g (*n* = 4) and 290–300 g (*n* = 4). Rats within each block were then sequentially numbered from 1 to 28. Subsequently, rats within each block were allocated to the different experimental groups using a random number table. Group I: defects without treatment (Mod group), Group II: defects treated with Gelatin methacryloyl (GelMA group), Group III: defects transplanted with iMSC/oeNC loaded GelMA (iMSC/oeNC group), and Group IV: defects transplanted with iMSC/oeFKBP5 loaded GelMA (iMSC/oeFKBP5 group). To perform transplantation, the rats were anaesthetised with 50 mg/kg ketamine and 5 mg/kg xylazine hydrochloride and then shaved. Then, critical‐sized defects (8 mm in diameter) were created at the central area of the cranium using a trephine.

Because normal SD rats (not immune deficient rats) were used in our transplantation experiment, to exclude the immune rejection effect of allogeneic human cell transplantation to animals, immunosuppressant cyclosporine A was routinely used post‐surgery [35]. After transplantation, the incision was stitched. Immunosuppressant cyclosporine A (59865‐13‐3, Sigma, USA) was injected at the dose of 5 mg/kg per day in rats. Since equivalent doses of cyclosporine A were administered to mice of all experimental groups concurrently, this effect does not compromise the reliability of the experiment.

### 2.11 Micro‐Computed Tomography

The rats were anaesthetised and scanned with micro‐computed tomography (micro‐CT, SkyScan1276, Bruker, Belgium) at week 6 and week 10 after surgery, respectively. Three‐dimensional (3D) reconstruction was applied by the multimodal 3D visualisation software (Siemens, Germany). The new bone volume fraction (bone volume/tissue volume, BV/TV), bone mineral density (BMD), trabecular number, and trabecular spacing were all calculated by CTAn 1.5 (Bruker, Belgium).

### 2.12 Histological Evaluation

At week 10 after transplantation, the animals were sacrificed by CO_2_ inhalation, and the calvarial defect sites were fixed for 48 h in 4% paraformaldehyde. They were then preserved in 10% neutral buffered formalin for an overnight period before being utilised for decalcification with 10% EDTA for a week. The tissue blocks were sectioned into 5 μm slices. Histological evaluation was performed with haematoxylin and eosin and Masson staining. Before staining according to a conventional protocol, tissue sections were rehydrated by a repeated xylene/ethanol/water rehydration procedure.

### 2.13 Statistical Analysis

Data was statistically analysed using SPSS 26.0 and plotted with GraphPad Prism 8. All data are given as the mean ± SEM for parametric and median and interquartile range for nonparametric data. For two‐group comparisons, homoscedasticity was determined by *F*‐test. Homoscedastic data were examined using Student's *t*‐test, while heteroscedastic data were analysed using Welch's *t*‐test. For multi‐group comparisons, homoscedasticity was evaluated by Brown–Forsythe test. Homoscedastic data underwent one‐way ANOVA, while heteroscedastic data were analysed using Welch's ANOVA. Statistical significance was assessed using Tukey's multiple comparison post hoc test. For all analyses, differences were considered statistically significant when *p* values were < 0.05.

## 3 Results

### 3.1 FKBP5 Was Dramatically Elevated During the Osteogenesis Process of iMSCs

**FIGURE 1:**
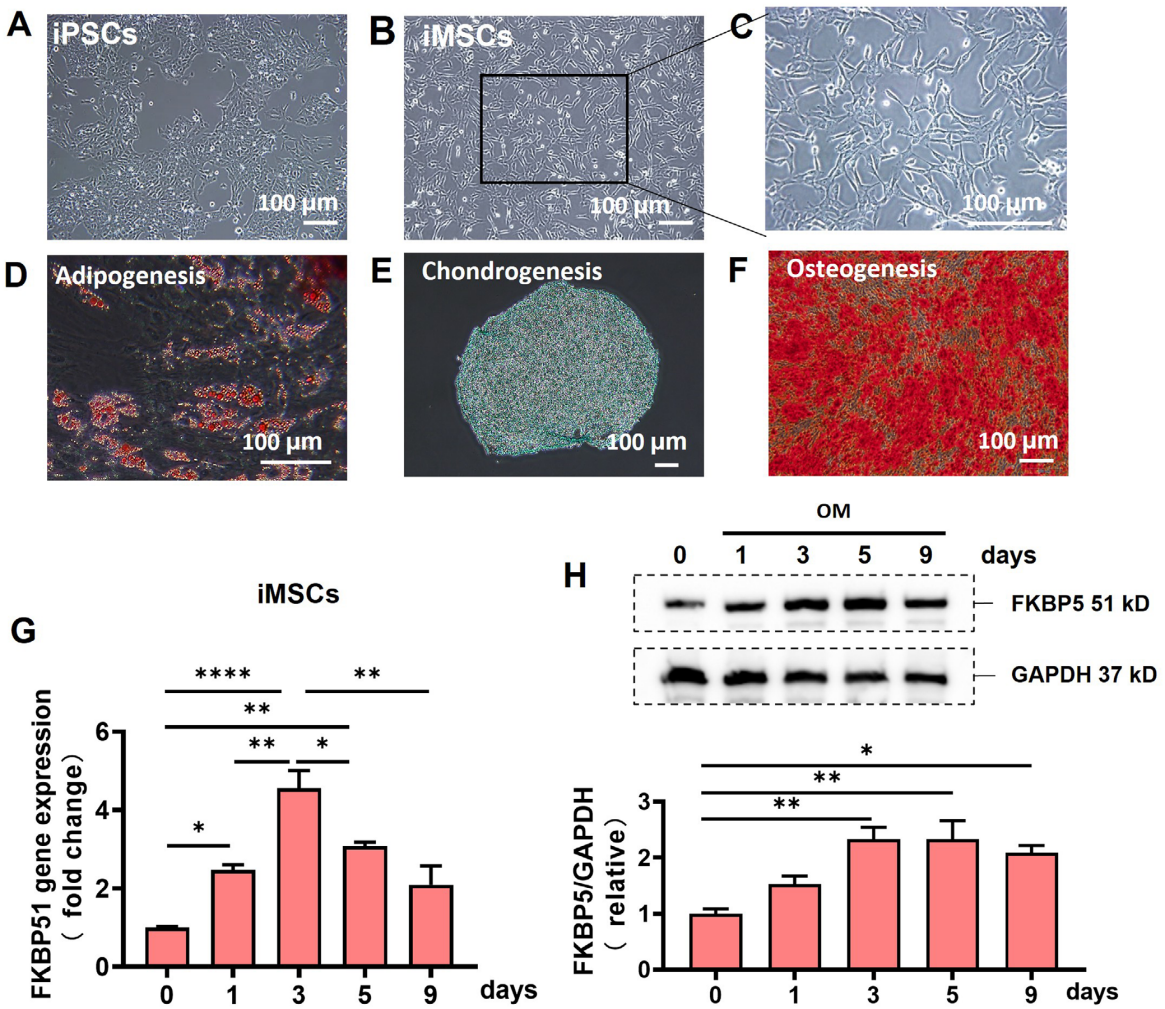
The expression of FKBP5 increased during osteogenesis in iMSCs. (A–C) IPSCs were induced into iMSCs; the image of C is a zoomed version of B. (D–F) The multilineage differentiation potential of iMSCs was examined, showing their ability to differentiate into adipocytes demonstrated by positive Oil Red O staining (D), chondrocytes demonstrated by positive Alcian Blue staining (E), and osteocytes demonstrated by positive Alizarin Red staining (F). (G) FKBP51 gene expressions at different time points after osteogenesis induction in iMSCs were detected by RT‐qPCR experiments. (H) FKBP5 protein expressions at different time points after osteogenesis induction in iMSCs were detected by Western blot, with the quantification result shown in the lower panel. FKBP, FK506 binding protein; iMSCs, Induced pluripotent stem cells derived mesenchymal stem cells. One‐way ANOVA analysis; Data are expressed as means ± SEM for three independent cell culture preparations per time point; *p < 0.05; **p < 0.01; ****p < 0.0001.

iMSCs were generated from iPSCs with the induction medium kit (RP01013, Nuwacell, China). Before induction, iPSCs exhibited a round, flat, and uniform morphology (Figure 1A). However, after induction, the cells stuck to the plastic culture flask in a configuration like a polygon and fibroblast (Figure 1B,C). The induced cells were collected for flow cytometry analysis (Figure S1). Flow cytometry analysis of iMSCs revealed high expression of typical MSC surface markers, including CD73 (98.70%), CD90 (99.90%), and CD105 (95.30%). In contrast, the expression of HLA‐DR (0.096%), CD34 (0.50%), CD14 (0.63%), CD45 (0.36%) and CD19 (1.90%) was negligible. Multilineage differentiation potentials were evidenced based on positive staining of Oil Red O for adipocytes (Figure 1D), Alcian Blue for chondrocytes (Figure 1E), and Alizarin Red for osteocytes (Figure 1F), respectively. The data above evidenced that these induced cells from iPSCs reach the criteria of MSCs [36], which means that iMSCs can be successfully generated from iPSCs.

Firstly, we examined the FKBP5 expression status in iMSCs under osteogenic induction. Gene FKBP51 expression significantly increased in iMSCs on day 1 after induction, and it reached the peak on day 3 at mRNA levels detected by RT‐qPCR (Figure 1G), and protein levels detected by western blot (Figure 1H). These results hinted that FKBP5 may play a role in the osteogenic development of iMSCs.

### 3.2 The Down‐Regulation of FKBP5 Leads to a Decrease in the Osteogenic Potential of iMSCs

**FIGURE 2:**
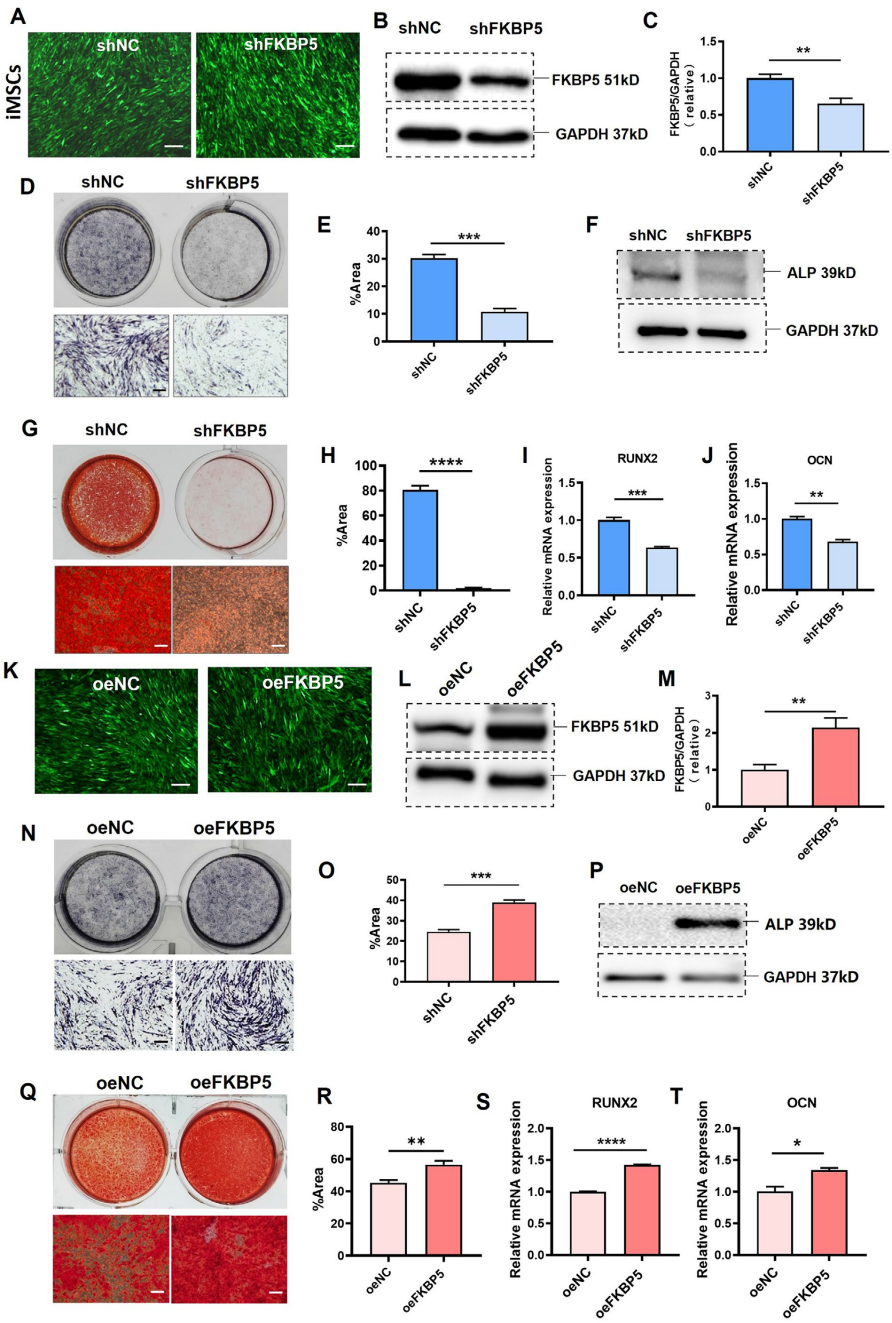
FKBP5 knockdown down‐regulated the osteogenesis of iMSCs. (A) IMSCs infected by knockdown or control lentivirus (MOI = 30) were grouped as iMSC/shFKBP5 and iMSC/shNC, respectively. The green fluorescence can be seen in iMSCs 48 h after transfection. (B, C) FKBP5 protein expression levels were detected by Western blot at 48 h after transfection (B); quantification of FKBP5 expression was analysed by ImageJ densitometry (C). (D) ALP expression was examined by ALP staining after 7 days of osteogenic induction. The upper panel shows gross images, while the lower panel shows microscopic images. (E) The percentages of ALP positive area (blue) in (D) were calculated by ImageJ software. (F) The ALP protein expressions were also detected by Western blot. (G) ARS experiments were performed after 14 days' culture under an osteogenic medium. The upper panel shows gross images while the lower panel shows microscopic images. (H) The percentages of positive area (red) in (G) were calculated with ImageJ software. (I, J) The expressions of RUNX2 (I) and OCN (J) at the mRNA level were examined using RT‐qPCR after osteogenic induction. (K) IMSCs infected by overexpression or control lentivirus (MOI = 30) were grouped as iMSC/oeFKBP5 and iMSC/oeNC, respectively. The green fluorescence can be seen in iMSCs 48 h after transfection. (L, M) FKBP5 protein expression levels were detected by Western blot at 48 h after transfection (L); Quantification of FKBP5 expression was analysed by ImageJ densitometry (M). (N) ALP expression was examined by ALP staining after 7 days' culture under an osteogenic medium. The upper panel shows gross images while the lower panel shows microscopic images. (O) The percentages of ALP positive area (blue) in (N) were calculated by ImageJ software. (P) The ALP protein expressions were also detected by Western blot. (Q) ARS experiments were performed after 14 days of culture under an osteogenic induction medium. The upper panel shows gross images while the lower panel shows microscopic images. (R) The percentages of positive area (red) in (Q) were calculated by ImageJ software. (S, T) The expressions of RUNX2 (S) and OCN (T) at the mRNA level were examined using RT‐qPCR after osteogenic induction. ALP, Alkaline phosphatases; ARS, Alizarin red staining; FKBP, FK506 binding protein; iMSCs, Induced pluripotent stem cells derived mesenchymal stem cells; iPSCs, Induced pluripotent stem cells; OCN, osteocalcin; RUNX2, Runt‐related transcription factor 2. Bar = 100 μm. The *F*‐test demonstrated homoscedasticity. Student's *t*‐test; Data are expressed as means ± SEM for three biological repeats of independent cell culture preparations per group; *p < 0.05; **p < 0.01; ***p < 0.001; ****p < 0.0001.

To determine the role in the osteogenesis process of iMSCs, FKBP5 was knocked down in the iMSCs utilising shRNA lentivirus (iMSCs/shFKBP5) or control lentivirus (iMSCs/shNC) (Figure 2A). Western blot analysis confirmed the successful suppression of FKBP5 protein in the iMSCs/shFKBP5 group (Figure 2B,C). Subsequently, two groups of cells were cultured in an osteogenic differentiation medium. After 7 days of osteogenic induction, ALP in the iMSCs/shFKBP5 group was significantly suppressed compared with the iMSCs/shNC group as revealed by ALP staining (Figure 2D,E). ALP protein level was also suppressed accordingly (Figure 2F). After 14 days of osteogenic induction, Alizarin red staining results showed a notable suppression in mineralisation of the iMSCs/shFKBP5 group (Figure 2G,H). Runt‐related transcription factor 2 (Runx2) is a fundamental transcription factor for bone development. Osteocalcin (OCN) is the most abundant non‐collagenous and osteoblast‐secreted protein in bone. Furthermore, the expressions of RUNX2 and OCN were significantly reduced in the iMSCs/shFKBP5 group (Figure 2I,J). The inhibition of osteogenesis was also observed in iMSCs treated with the selective FKBP5 inhibitor SAFit2 (Figure S2). These findings collectively indicate that the down‐regulated expression or functional inhibition of FKBP5 hinders the osteogenic differentiation of iMSCs.

### 3.3 The Overexpression of FKBP5 Enhanced the Osteogenic Potential of iMSCs

To further validate the role of FKBP5 in iMSCs, FKBP5 was also overexpressed using lentivirus infection (Figure 2K). The efficiency was determined through western blot analysis (Figure 2L,M). The iMSCs then underwent osteogenic differentiation. Staining for ALP showed a significant enhancement in the iMSCs/oeFKBP5 group compared to the iMSCs/oeNC group at D7 after induction (Figure 2N,O). The up‐regulation of ALP was also observed at protein levels (Figure 2P). At D14 after induction, the mineralisation of the iMSCs/oeFKBP5 group was significantly enhanced, as confirmed by Alizarin red staining (Figure 2Q,R). Additionally, the expressions of RUNX2 and OCN were considerably up‐regulated (Figure 2S,T). These results provide evidence that elevated FKBP5 expression promotes osteogenic differentiation.

### 3.4 Protein Variances Between iMSCs/oeFKBP5 and iMSCs/oeNC During Osteogenesis

**FIGURE 3:**
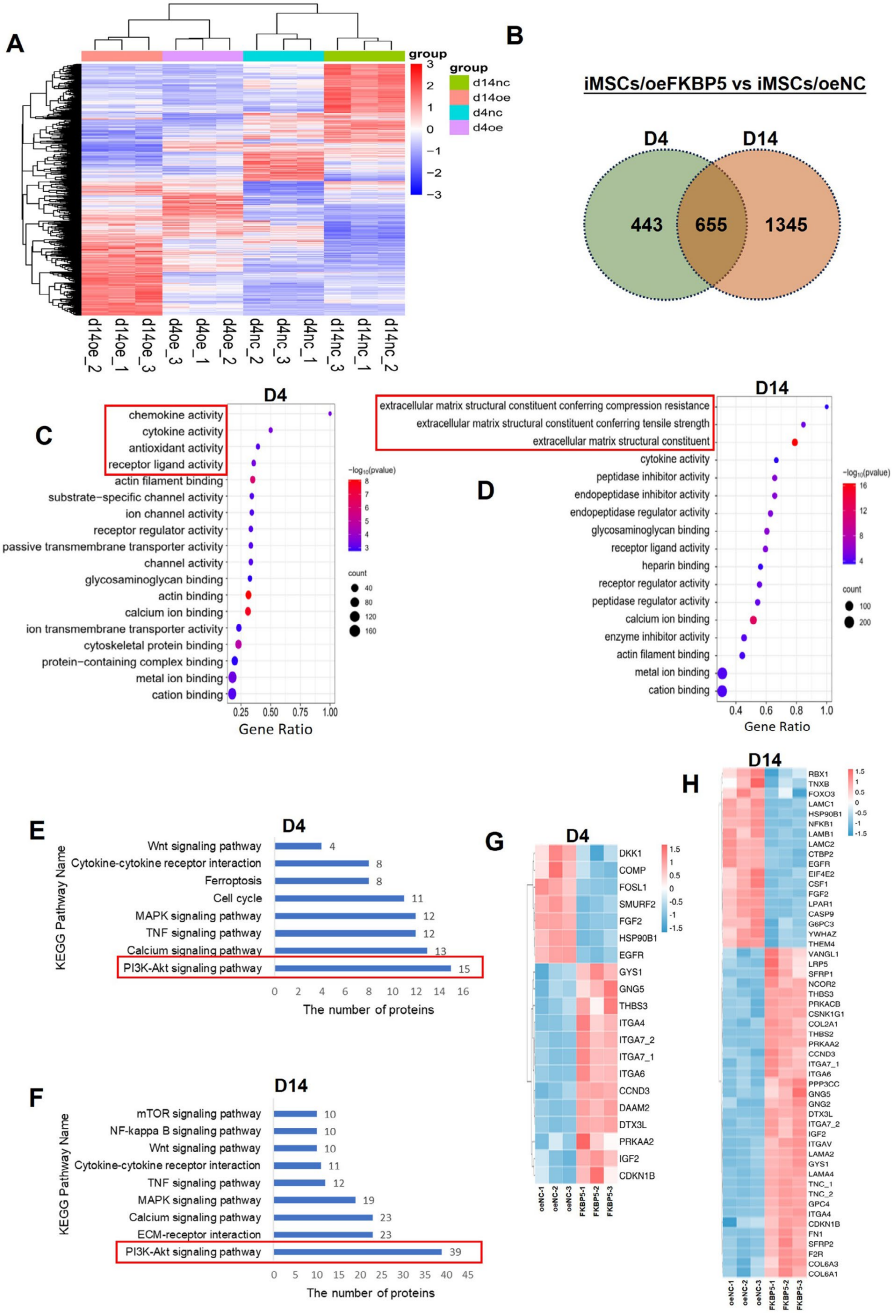
Proteomics detection of iMSCs/oeFKBP5 and iMSCs/oeNC after osteogenic induction. (A) Protein expressions of iMSCs/oeFKBP5 and iMSCs/oeNC group at D4 and D14 after osteogenic induction, and the results were presented in the form of Heatmap. (B) The Veen map of the differential proteins in two groups of cells on D4 and D14. (C, D) The bubble charts of the enrichment of GO entries at D4 (C) and D14 (D) after induction. The size of the coloured rings represents the amount of protein enrichment. (E, F) KEGG analysis of the osteogenesis‐related cell signal in the differential proteins between the two groups of iMSCs at D4 (E) and D14 (F). (G, H) The heatmap exhibition of differential proteins involved in the osteogenesis‐related cell signals at D4 (G) and D14 (H). GO, Gene Ontology; KEGG, Kyoto Encyclopedia of Genes and Genomes.

To investigate the molecular basis of enhanced osteogenesis by FKBP5, comprehensive proteomics analysis was carried out on D4 and D14 after osteogenic induction. At D4, 438 proteins were down‐regulated and 660 proteins were up‐regulated in iMSCs/oeFKBP5 compared to the control iMSCs/oeNC group. At D14, 899 proteins were down‐regulated, and 1101 proteins were up‐regulated. According to cluster analysis of differential proteins, the expression patterns of protein files in iMSCs/oeFKBP5 and iMSCs/oeNC differed extensively (Figure 3A). The Venn diagram indicated that most differential proteins at D4 and D14 were distinct, and the impact of FKBP5 on cells increased with time (Figure 3B). The bubble chart of GO analysis showed the significantly changed proteins were enriched for multiple molecular activities at D4 and D14 (Figure 3C,D). On D4, the differential proteins were primarily involved in intracellular functions, such as actin‐binding and calcium binding, while at D14, the significantly changed proteins were enriched in extracellular matrix functions. According to KEGG analysis (Figure 3E,F), the differential proteins were enriched in several osteogenesis‐related signalling pathways, including PI3K‐AKT, Wnt, MAPK, and calcium signalling pathways. It was noteworthy that the differential protein enrichment of the PI3K‐AKT signalling pathway is highest at both D4 and D14. The heatmaps displayed the significantly changed proteins related to osteogenic differentiation at D4 (Figure 3G) and D14 (Figure 3H). These data lay the molecular basis of enhanced osteogenesis by FKBP5.

### 3.5 FKBP5 Enhanced the Osteogenesis of iMSCs via FKBP5‐AKT‐FOXO1 Pathway

**FIGURE 4:**
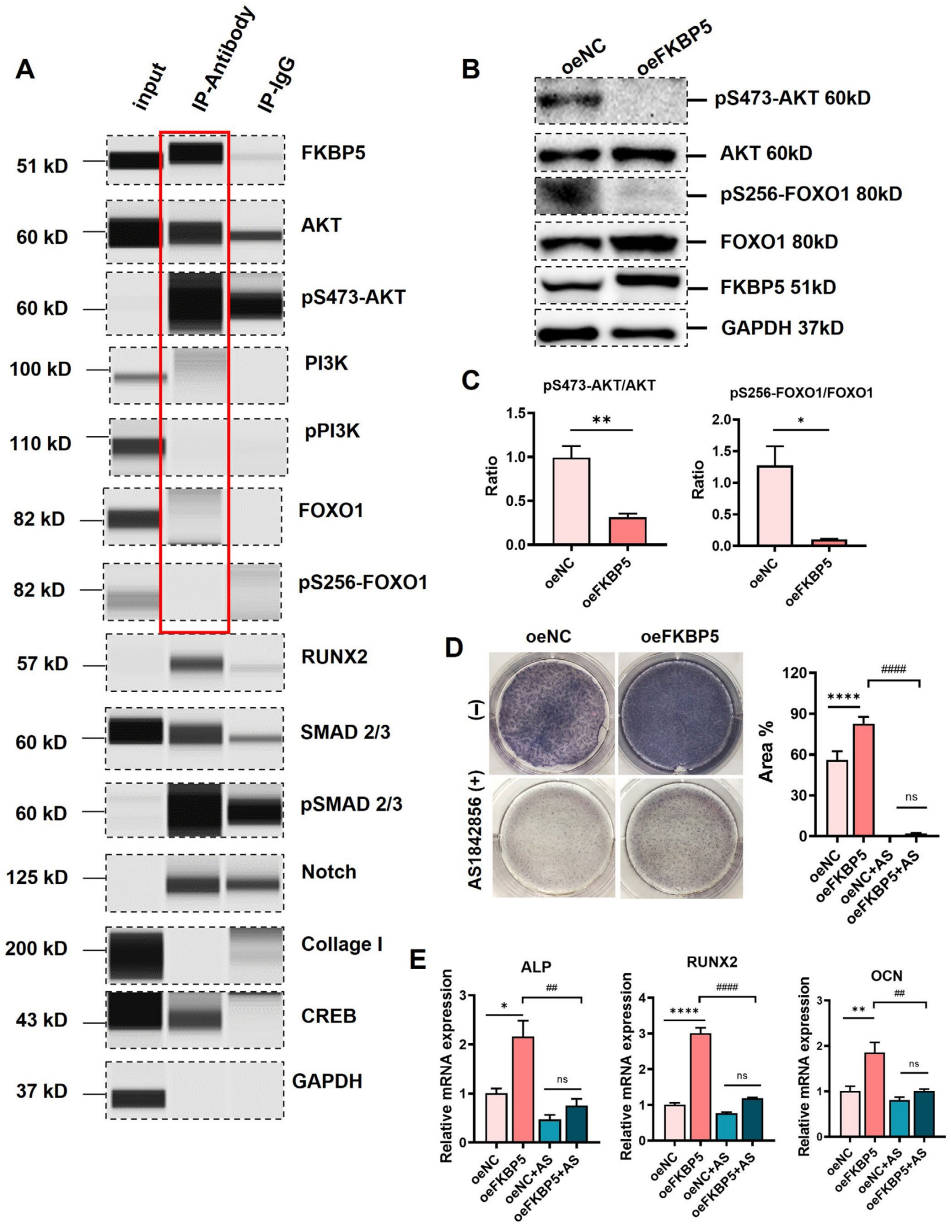
FKBP5 enhanced the osteogenesis of iMSCs via the FKBP5‐AKT‐FOXO1 pathway. (A) The binding proteins of FKBP5 detected by the co‐IP technique in iMSCs. (B) The expression status of pS473‐AKT, AKT, pS256‐FOXO1 and FOXO1 in the iMSCs/oeNc and iMSCs/oeFKBP5. (C) The quantification of pS473‐AKT/AKT, pS256‐FOXO1/FOXO1 in the two groups of cells. The *F*‐test demonstrated homoscedasticity. Student's *t*‐test; data are expressed as means ± SEM for three independent cell culture preparations per group. (D) The FOXO1 inhibitor AS1842856 (1 μM) was added to the osteogenic induction culture medium with an equal volume of vehicle (DMSO) added as a negative control. The ALP staining was carried out at D7 after induction with the quantification result of the positive area (blue) according to ImageJ software shown in the right panel. The Brown–Forsythe test demonstrated homoscedasticity. One‐way ANOVA analysis; Data are expressed as means ± SEM for three independent cell culture preparations per condition. (E) The expression levels of osteogenesis‐related gene ALP, OCN, RUNX2 were also detected. The Brown–Forsythe test demonstrated homoscedasticity. One‐way ANOVA analysis: Data are expressed as means ± SEM for three independent cell culture preparations per condition. ALP, Alkaline phosphatases; FKBP, FK506 binding protein; iMSCs, Induce pluripotent stem cells derived mesenchymal stem cells; OCN, osteocalcin; RUNX2, Runt‐related transcription factor 2. *p < 0.05 vs. oeNC; **p < 0.01 vs. oeNC; ***p < 0.001 vs. oeNC; ****p < 0.0001 vs. oeNC; ^##^ *p* < 0.01 vs. oeFKBP5; ^####^ *p* < 0.0001 vs. oeFKBP5.

Considering that FKBP5 is a co‐chaperone for various proteins, and that the differential protein enrichment of D4 and D14 is primarily focused on the PI3K‐AKT signalling pathway, the binding proteins of FKBP5 in iMSCs were detected by the co‐IP technique. The results showed that AKT and pS473‐AKT are binding proteins of FKBP5 in iMSCs, but not PI3K, p‐PI3K (upstream proteins of AKT), and FOXO1, pS256‐FOXO1 (downstream proteins of AKT) (Figure 4A). Further biological experiments showed that FKBP5 overexpression in iMSCs decreased the ratios of pS473‐AKT/AKT and pS256‐FOXO1/FOXO1 when compared with those of iMSCs/oeNC (Figure 4B,C). In line with earlier findings, FOXO1 is a direct target of AKT and plays a key role in osteogenic differentiation [37]. AKT‐pS473 can phosphorylate FOXO1 at Ser256, leading to further degradation in the cytoplasm [38]. To confirm the crucial role of FOXO1 in the osteogenesis in iMSCs, the FOXO1 inhibitor AS1842367 (1 μM) was applied in the osteogenic induction culture medium. At D7 after induction, AS1842367 application dramatically decreased the ALP positive area of cells in the iMSCs/oeFKBP5 group and iMSCs/oeNC group, and the difference between the two groups disappeared (Figure 4D); the gene expressions of ALP, RUNX2 and OCN changed accordingly (Figure 4E), suggesting that FOXO1 inhibition hampered the enhanced osteogenesis of iMSCs by FKBP5. The results above indicated that the FKBP5‐AKT‐FOXO1 pathway plays a crucial role in the osteogenic differentiation of iMSCs.

### 3.6 Delivery of FKBP5 Overexpressed iMSCs Accelerated the Healing of Critical‐Sized Calvarial Defect In Vivo

**FIGURE 5:**
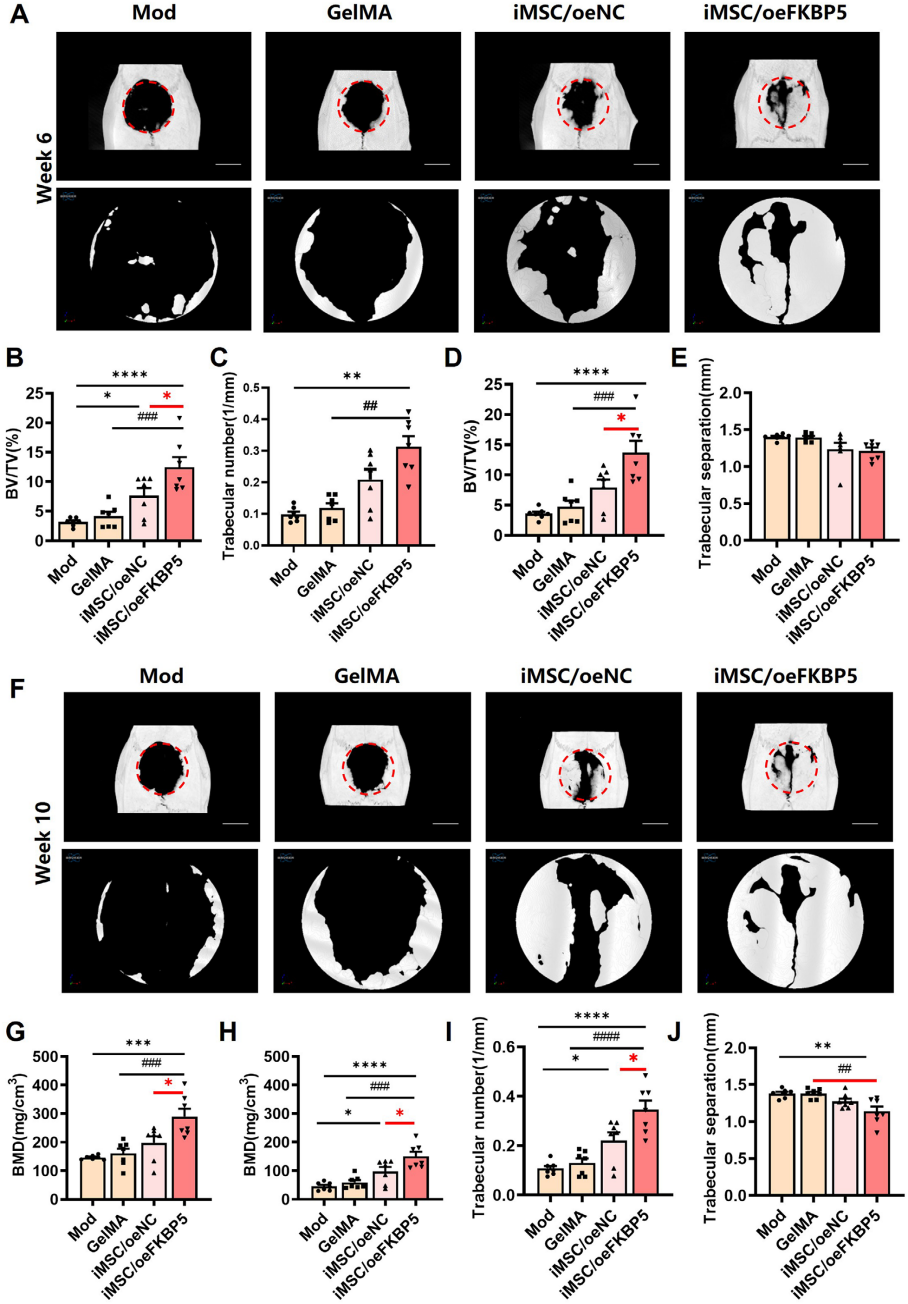
Micro‐CT analysis of bone regeneration post‐transplantation. (A–E) At week 6 after the transplantation, micro‐CT was carried out to investigate the regeneration status of the calvarial defects. Reconstructed 3D micro‐CT images of bone formation were gained at week 6 (A) and week 10 after transplantation (F). New bone volume fraction (BV/TV) (B), BMD (C), trabecular number (D), and trabecular spacing (E) were all calculated. (F–J) At week 10 after the transplantation, the reconstructed 3D micro‐CT images (F) and the corresponding parameters were also calculated (G–J). BMD, bone mineral density; BV, bone volume; TV, tissue volume. Except for (C), which violated homoscedasticity and was analysed using Welch ANOVA, all other groups met the assumption of homogeneity of variance and were subjected to one‐way ANOVA; data are represented as mean ± SEM obtained in seven rats per condition; *p < 0.005; **p < 0.01; ***p < 0.001; ****p < 0.0001 versus Mod group. ##*p* < 0.01, ###*p* < 0.001, ####*p* < 0.0001 versus GelMA group. Bar = 4mm.

To investigate the role of FKBP5 in iMSCs in an in vivo setting, a rat critical‐sized calvarial defect model was established (Figure S3A). The surgery process is shown in Figure S3B. Biomaterial GelMA loaded with or without iMSCs was delivered to the defect locations. The healing capacity was assessed through continuous observation using micro‐CT imaging at week 6 and 10 post‐surgery. There are no exclusions of animals or data points during the analysis (*n* = 7 for each group). The micro‐CT images showed that the defect size remained unchanged in the model group (Figure 5A,F, Mod group) and marginally contracted in the GelMA transplanted group (Figure 5A,F, GelMA group). In contrast, both iMSCs/oeNC group and iMSCs/oeFKBP5 group showed a smaller defect size, with the iMSCs/oeFKBP5 group being more effective at week 6 post‐surgery (Figure 5A). Interestingly, at week 10 post‐surgery, the iMSCs/oeFKBP5 group exhibited a larger radio‐opaque mass, representing newly formed mineralised bone tissue, and almost complete closure of the defect was observed (Figure 5F, the fourth column).

**FIGURE 6:**
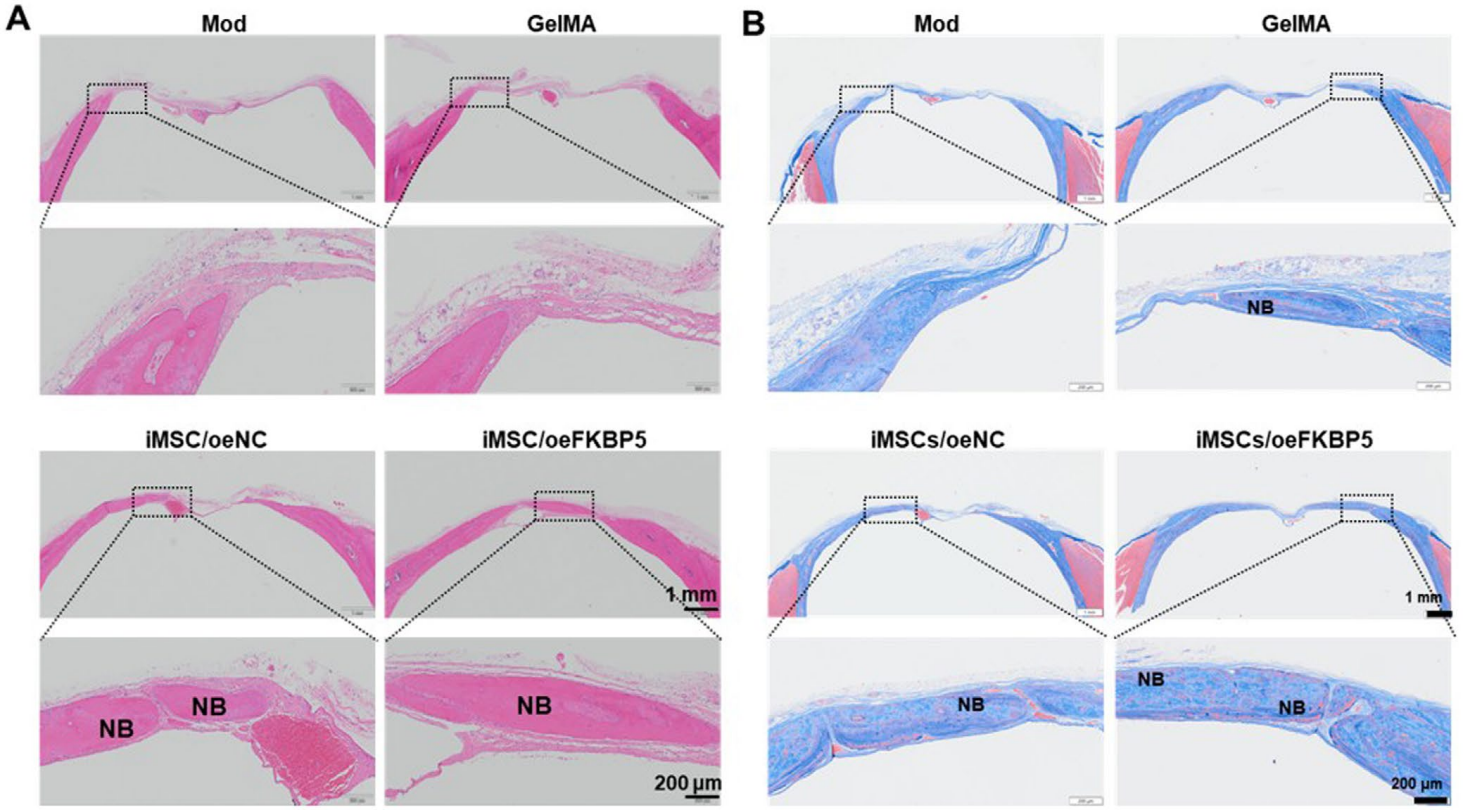
Histological evaluations of bone formation at week 10 post‐transplantation. (A) H&E staining was conducted at week 10 post‐transplantation. (B) Characterisation of collagen deposition evaluated by Masson staining. Muscle fibres are stained red, collagen fibres and immature bone tissue are stained blue, and mature bone tissue is light red. H&E, haematoxylin and eosin; NB, new bone.

Further evaluation of the bone defects revealed that the new bone volume fraction (BV/TV), BMD, and trabecular number were significantly increased in the iMSCs/oeFKBP5 group compared to the iMSCs/oeNC group at both week 6 (Figure 5B–D) and week 10 (Figure 5G–I). The trabecular spacing was significantly decreased at week 10 post‐surgery in the iMSCs/oeFKBP5 group (Figure 5J). To observe the dynamic repair process of each animal, we only conducted the histological examination of the bone defect at week 10. Neo‐tissue formations were observed in all the groups except the Mod group according to H&E staining. Interestingly, the iMSCs/oeFKBP5 group showed significant tissue regeneration and almost osseous closure (Figure 6A). Characterisation of collagen deposition was evaluated by Masson staining. Muscle fibres are stained red, collagen fibres and immature bone tissue are stained blue, and mature bone tissue is light red (Figure 6B). For the Mod group, little collagen deposition can be observed at the edge of the defect. For the iMSCs/oeFKBP5 group, dense collagen deposition was observed in the defect, with most of the defect filled with collagen. These results confirmed the increased deposition of new bone in the iMSCs/oeFKBP5 group compared to the iMSCs/oeNC group.

## 4 Discussion

Given that iMSCs serve as a valuable source for tissue engineering transplantation, FKBP5 is a molecular chaperone and acts as a scaffolder of various protein complexes engaged in signal transduction, but what effects FKBP5 has in the osteogenesis process of iMSCs remains unknown. The results of our study demonstrated that FKBP5 promoted osteogenic differentiation, suggesting potential new strategies for iMSCs transplantation in the clinical treatment of bone defects.

In the late 1980s, Siekierka and Harding's group discovered a novel protein, FK506 binding protein (FKBP) [39]. FKBP5, encoded by the gene of FKBP51, was found to be a 51‐kDa immunophilin belonging to the high molecular weight immunophilin of the FKBP family. FKBP5 exerts its functions in two ways: one is to bind with proteins as molecular chaperones, and the other is to act as protein scaffolds and participate in signal transduction due to its unique molecular structure. As most reported studies about FKBP5 on Pubmed are related to energy metabolism, cell proliferation and cancer, stress‐related diseases, and phenotypes, immune function [40], FKBP5 in clinical studies was focused on stress‐related psychiatric diseases. Common allelic variants in the FKBP51 gene are associated with an increased risk of developing affective disorders like anxiety, depression, schizophrenia, and post‐traumatic stress disorder [14]. Therefore, FKBP5 knockout mice have been extensively applied in related studies. FKBP5 KO mice reduced HPA activity and showed no response to acute stress. There was a rarely reported study about the relationship between bone defect and KO mice. Additionally, FKBP5 has been implicated in cell differentiation. Kikyo N reported that FKBP5 promoted myoblast differentiation via Cdk4 isomerization [41, 42]. Using FKBP5^V55L^ mice, Zhao's group reported that FKBP5 mutation increased the osteoclast genesis of the osteoclast precursors due to their hyperresponsiveness to RANKL, and increased the osteoclasts' function due to the greater bone‐resorbing activity [34]. This information suggested that FKBP5 may play a critical role in bone metabolism, which is a balance of osteogenesis and osteoclasis, as well as the result of whole and balanced regulation of the internal environment of the body. This is an interesting point worth further exploring in depth.

So far, there is no reported study on the role and mechanism of FKBP5 in iMSC osteogenesis. FKBP5 increased after osteogenesis in iMSCs (Figure 1). Therefore, we selected FKBP5 as our target molecule to explore its role as either an enhancer, inhibitor, or neutral factor in the osteogenesis process of iMSCs, as well as the possible underlying mechanism. Next, we used lentivirus to modulate the expression of FKBP5. Our results suggest that the down‐regulation of FKBP5 hinders osteogenic differentiation while the overexpression of FKBP5 promotes the process (Figure 2). These findings support the notion that FKBP5 can serve as a biomarker for MSC osteogenesis. It is of note that the expression of FKBP5 also increases not only during the process of osteogenesis but also during chondrogenesis and adipogenesis of MSCs as previously reported. FKBP5 may enhance the differential potential of iMSCs, but not selectively the osteogenesis of iMSCs.

To elucidate the molecular basis underlying the effects of FKBP5 on osteogenic differentiation, a proteomic analysis was conducted to identify significant differences in protein expression between iMSCs overexpressing FKBP5 and control iMSCs. The results revealed notable disparities in the expression profiles of the two groups. KEGG enrichment analysis demonstrated that the differential proteins were enriched in osteogenesis‐related pathways, including the PI3K‐AKT signalling pathway, Wnt signalling pathway, etc. The highly enriched PI3K‐AKT pathway provided valuable insights into the regulatory role of FKBP5 in osteogenesis (Figure 3). Then our following investigations focused on the interaction of FKBP5 and proteins involved in the PI3K‐AKT pathway.

It is reported that FKBP5 has a negative regulatory effect on AKT activity, while FKBP5 can inhibit AKT activation by enhancing Ser473 dephosphorylation. Studies have shown that FKBP5 can function as a scaffold protein to enhance PHLPP‐AKT interaction, leading to PHLPP‐mediated pS473‐AKT dephosphorylation [43]. Down‐regulation of FKBP5 leads to a decrease in PHLPP‐AKT interaction, resulting in an increase in pS473‐AKT phosphorylation [44]. Conversely, overexpression of FKBP5 leads to a decrease in pS473‐AKT phosphorylation [45, 46]. However, our investigation into downstream proteins in the AKT pathway revealed a decrease in FOXO1 phosphorylation, which may promote osteogenic differentiation. FOXO1 is a direct target regulated by AKT and a key regulator of osteogenic differentiation [47]. pS473‐AKT can phosphorylate FOXO1 at Ser256, leading to further degradation in the cytoplasm [23, 37]. Reports have indicated that FOXO1 promotes osteogenic differentiation in MSCs [48, 49, 50], and knockdown of FOXO1 reduces the expression of osteogenic‐specific OCN and OPN in MSCs after osteogenic induction [50]. In contrast, the FOXO1 agonist 2‐Fly protects the osteogenic potential of periodontal ligament stem cells and promotes bone formation under inflammatory conditions [49]. Chromatin immunoprecipitation analysis showed that FOXO1 directly interacts with the RUNX2 promoter, thereby increasing the transcription and expression of the osteogenic‐related gene RUNX2 [51]. To further verify this, biological experiments were conducted here to examine the role of FOXO1 in the osteogenic differentiation of iMSCs. AS1842856 is a cell‐permeable inhibitor that can directly bind to activated FOXO1, but not to pS256‐FOXO1 [52]. The application of AS1842856 weakened the promoting effect of FKBP5 on the osteogenic differentiation of iMSCs, indicating that FKBP5 can promote the osteogenic differentiation of iMSCs by regulating FOXO1. In summary, FOXO1 is an important target for FKBP5 to control the osteogenic differentiation of iMSCs. Overexpression of FKBP5 can increase AKT and dephosphorylate pS473‐AKT, leading to a decrease in pS256‐FOXO1 and a reduction in its intracellular degradation, resulting in an increased function. Our study confirms for the first time that the FKBP5‐AKT‐FOXO1 pathway plays a critical role in promoting the osteogenic differentiation of iMSCs (Figure 4). It is worth noting that FKBP5 acts as a molecular chaperone for multiple proteins; the molecules above (RUNX2, SMAD, Notch, CREB) are all important for the osteogenesis process of MSCs. The modifications in various cellular signalling pathways in iMSCs may not be excluded for the underlying molecular mechanisms of this process.

In our study, a rat model of an 8 mm‐diameter critical‐sized bone defect was made to determine whether the transplantation of biomaterial‐loaded iMSCs/oeFKBP5 would have an enhanced bone regeneration effect in vivo (Figures 5 and 6). Compared with the GelMA and GelMA loaded with iMSCs/oeNC transplantation groups, the GelMA loaded with iMSCs/oeFKBP5 delivery was particularly effective, as demonstrated by micro‐CT and histological staining. These findings are consistent with previous studies demonstrating the beneficial effects of iMSCs in bone regeneration in mini‐pigs [53], and in a radius of nude mice [54]. Our findings further proved that elevated FKBP5 expression could promote osteogenic differentiation in vivo. Considering the complexity of in vivo transplantation settings, we did not explore the crucial cell signal effect, which is worth further investigation.

## 5 Conclusion

**FIGURE 7:**
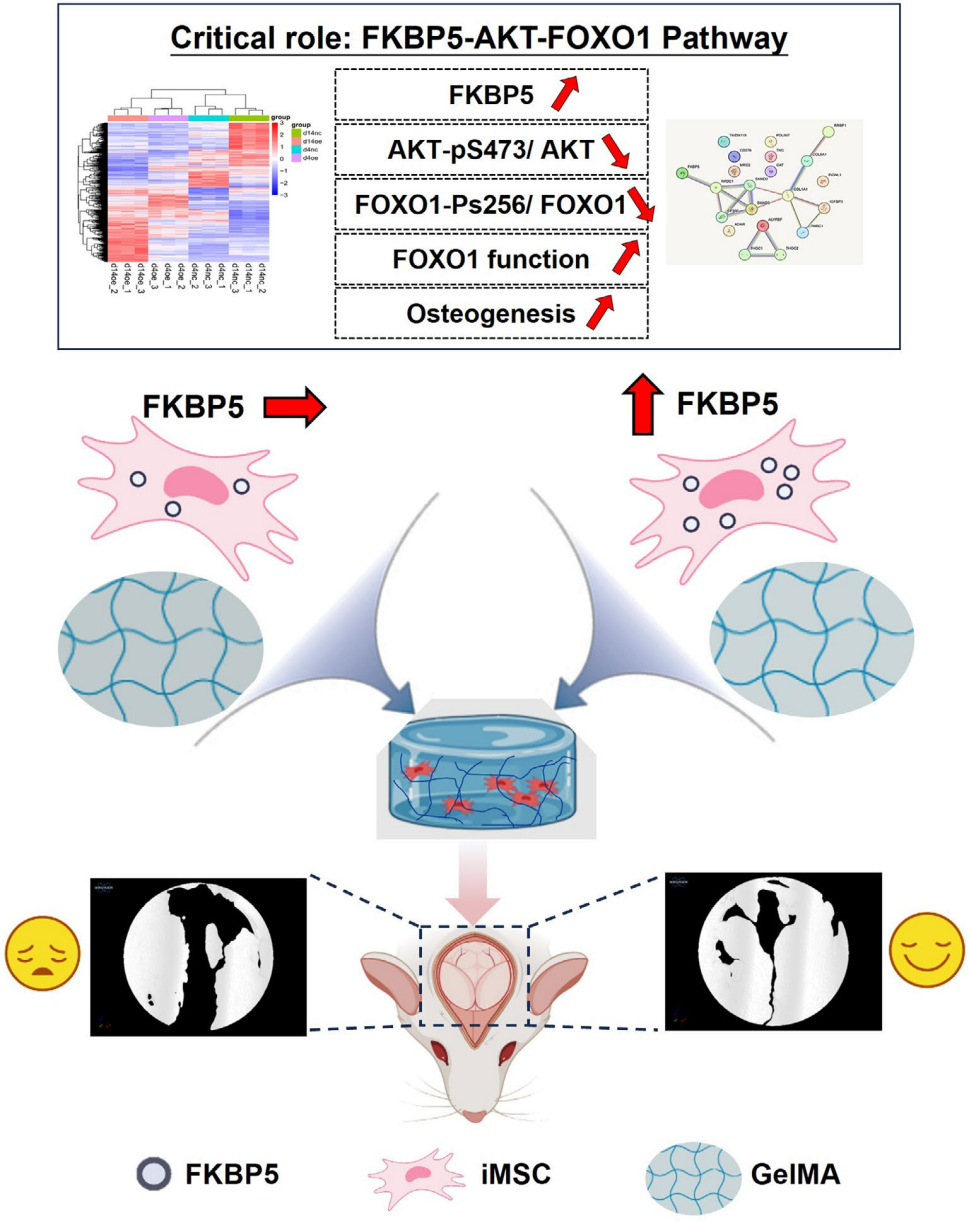
The working model of FKBP5 promotes osteogenesis of the transplanted iMSCs for bone defect treatment. FKBP5‐AKT‐FOXO1 pathway acts as a critical role in the osteogenesis process of iMSCs. FKBP5 decreases the ratios of AKT‐pS473/AKT and FOXO1‐pS256/FOXO1; the function of FOXO1 increases. Therefore, FKBP5‐overexpressed iMSCs are loaded in biomaterials and transplanted into the defect site of rats; the healing effects are enhanced in the calvarial defects.

Our research findings demonstrated that FKBP5 has a beneficial impact on iMSCs osteogenic differentiation, and the FKBP5‐AKT‐FOXO1 pathway plays a crucial role in the process. The application of FKBP5‐overexpressing iMSCs enhances the therapeutic effect of critical‐sized bone defects (Figure 7). Our findings contribute to understanding FKBP5's physiological function in osteogenesis, and provide a novel approach for the clinical management of bone defects. Furthermore, the transplantation potential of iMSCs/FKBP5 should be validated in multiple large animal models and clinical settings.

## Author Contributions

**Xiao‐Yu Tian:** investigation (lead), methodology (lead). **Biao Zhu:** data curation (lead), formal analysis (lead), investigation (equal). **Xiang‐Bin Zhou:** data curation (equal), formal analysis (equal), investigation (equal). **Wen‐Can Fang:** data curation (equal), investigation (equal). **Ye Lei:** investigation (supporting), methodology (supporting). **Meng‐Nan Liu:** data curation (equal), investigation (equal), methodology (supporting). **Ning Wu:** supervision (equal), writing – review and editing (equal). **Ning Wen:** data curation (equal), funding acquisition (equal), supervision (lead), writing – review and editing (lead). **Hong Li:** funding acquisition (lead), project administration (lead), writing – original draft (lead).

## Ethics Statement

Animal experiments involved were approved by the Institutional Animal Care and Use Committee of the Institute of Pharmacology and Toxicology, Beijing, China (Animal Ethics Approval No: IACUC‐DWZX‐2021‐666) and were conducted under the Guidelines for Animal Experimentation. The date of the ethics approval was on June 3, 2021.

## Conflicts of Interest

The authors declare no conflicts of interest.

## Supporting information


**Figure S1:** The phenotype of the cells induced from iPSCs was examined by Flow Cytometry.
**Figure S2:** Osteogenesis was restrained in iMSCs by the application of FKBP5 selective inhibitor SAFit2.
**Figure S3:** iMSCs transplantation in the rat model of critical‐sized calvarial defect.
**Table S1:** Antibodies used for cytometry flow.
**Table S2:** Three shRNA sequences for FKBP5 knockdown were desired.
**Table S3:** Primers used for real‐time qPCR.
**Table S4:** Antibodies used for Westen Blot.
**Table S5:** Antibodies used for co‐IP.

## Data Availability

The data that support the findings of this study are available from the corresponding author upon reasonable request.
