# Supplementary material for: FKBP5 Regulates Osteogenesis of Human iPSC‐Derived Mesenchymal Stem Cells via FKBP5‐AKT‐FOXO1 Pathway
Source: J Cell Mol Med. 2025 Oct 31;29(21):e70849. doi: 10.1111/jcmm.70849 (PMC12576591; doi:10.1111/jcmm.70849)
Supplement: Supplementary file 1 — Figure S1: The phenotype of the cells induced from iPSCs was examined by Flow Cytometry. Figure S2: Osteogenesis was restrained in iMSCs by the application of FKBP5 selective inhibitor SAFit2. Figure S3: iMSCs transplantation in the rat model of critical‐sized calvarial defect. Table S1: Antibodies used for cytometry flow. Table S2: Three shRNA sequences for FKBP5 knockdown were desired. Table S3: Primers used for real‐time qPCR. Table S4: Antibodies used for Westen Blot. Table S5: Antibodies used for co‐IP. [file JCMM-29-e70849-s001.zip › jcmm70849-sup-0001-supinfo.docx]

**Supplementary Figures：**

**Supplementary Figure 1. The phenotype of the cells induced from iPSCs was examined by Flow Cytometry.**

**Supplementary Figure 2. Osteogenesis was restrained in iMSCs by the application of FKBP5 selective inhibitor SAFit2.**

A: iMSCs were cultured in the osteogenesis induction medium with different concentrations of FKBP5 selective inhibitor SAFit2. Alizarin red staining was carried out at week 2 after induction.

B: The percentages of positive area (red) were calculated.

**Supplementary Figure 3. iMSCs transplantation in the rat model of critical-sized calvarial defect.**

A: The anatomic location of an 8 mm diameter calvarial defect on the rat’s skull.

B: Schematic diagrams of the 4 groups of rats involved in the study. Mod group, rats of only critical-sized calvarial defect; GelMA group, rats of GelMA transplantation into the model; iMSC/oeNC group, rats of iMSC/oeNC transplantation into the model; iMSC/oeFKBP5 group, rats of iMSC/oeFKBP5 transplantation into the model.

GelMA, Gelatin Methacryloyl.

C. The surgical process for establishing the rat model of critical-sized calvarial defect.

**Supplementary Tables：**

**Supplementary Table 1. Antibodies used for cytometry flow**

| Antibody | Cat.No | Company (Country) |
| --- | --- | --- |
| CD73-APC | 344005 | BioLegend (USA) |
| CD90-FITC | 328108 | BioLegend (USA) |
| CD34-PE | 378603 | BioLegend (USA) |
| CD14-PE | 325605 | BioLegend (USA) |
| CD19-FITC | 302206 | BioLegend (USA) |
| CD45-FITC | 304005 | BioLegend (USA) |
| CD105-APC | 562408 | BD Biosciences (USA) |
| HLA-DR-APC | 559866 | BD Biosciences (USA) |

**Supplementary Table 2. Three shRNA sequences for FKBP5 knockdown were desired**

| Code | **Plasmid** | **Sequence** |
| --- | --- | --- |
| LV-1309 | LV-U6-shRNA1(FKBP5)-CMV-mCherry-WPRE | GGTATGAAATCGACTCCTTAA |
| LV-1310 | LV-U6-shRNA2(FKBP5)-CMV-mCherry-WPRE | GCTTGAGCTCATGAACAAACA |
| LV-1311 | LV-U6-shRNA3(FKBP5)-CMV-mCherry-WPRE | GCATCCCACCTGCTCATATTC |
| LV-0144 | LV-U6-shRNA(scramble)-CMV-mcherry-T2A-Puro-WPRE(Control group） | CCTAAGGTTAAGTCGCCCTCG (None in human and mouse) |
| LV-1312 | LV-CMV-FKBP5-P2A-mcherry-WPRE (FKBP5 over-expression) | FKBP5 NM_001145775.3 human |

**Supplementary Table 3. Primers used for real-time qPCR**

| Gene | Forward primer | Reverse primer |
| --- | --- | --- |
| FKBP5 | AGGAGGGAAGAGTCCCAGTG | TGGGAAGCTACTGGTTTTGC |
| ALP | GCTTGACCTCCTCGGAAGACACTC | CGCCTGGTAGTTGTTGTGAGCATAG |
| RUNX2 | AGGCAGTTCCCAAGCATTTCATCC | TGGCAGGTAGGTGTGGTAGTGAG |
| OCN | AGGGCAGCGAGGTAGTGAAGAG | GGTCAGCCAACTCGTCACAGTC |
| GAPDH | ACCCAGAAGACTGTGGATGG | CACATTGGGGGTAGGAACAC |

**Supplementary Table 4. Antibodies used for Westen Blot**

| Antibody | Cat.No | Company (Country) |
| --- | --- | --- |
| FKBP5 | 12210 | Cell Signaling Technology (USA) |
| ALP | ab229126 | Abcam (UK) |
| β-actin | ab8227 | Abcam (UK) |
| GAPDH | IJ0417 | ZenBio (China) |

**Supplementary Table 5. Antibodies used for co-IP**

| Antibody | Cat.No | Company (Country) |
| --- | --- | --- |
| FKBP5 | 12210 | Cell Signaling Technology (USA) |
| AKT | 4691S | Cell Signaling Technology (USA) |
| P-AKT (Ser473) | 4060S | Cell Signaling Technology (USA) |
| SMAD2/3 | 8685T | Cell Signaling Technology (USA) |
| P-SMAD2/3 | AF3367 | Affinity Bioscience (USA) |
| FOXO1 | 2880 | Cell Signaling Technology (USA) |
| P-FOXO1 (Ser473) | 84192 | Cell Signaling Technology (USA) |
| CREB | 4820S | Cell Signaling Technology (USA) |
| PI3K | 3358T | Cell Signaling Technology (USA) |
| P-PI3K | 17366 | Cell Signaling Technology (USA) |
| Notch | ab52627 | Abcam (UK) |
| RUNX2 | ab23981 | Abcam (UK) |
| Collage I | 67288-1-Ig | Proteintech(USA) |
| GAPDH | IJ0417 | ZenBio (Chian) |
