# Supplementary figures and images for: FKBP5 Regulates Osteogenesis of Human iPSC‐Derived Mesenchymal Stem Cells via FKBP5‐AKT‐FOXO1 Pathway
Source: J Cell Mol Med. 2025 Oct 31;29(21):e70849. doi: 10.1111/jcmm.70849 (PMC12576591; doi:10.1111/jcmm.70849)

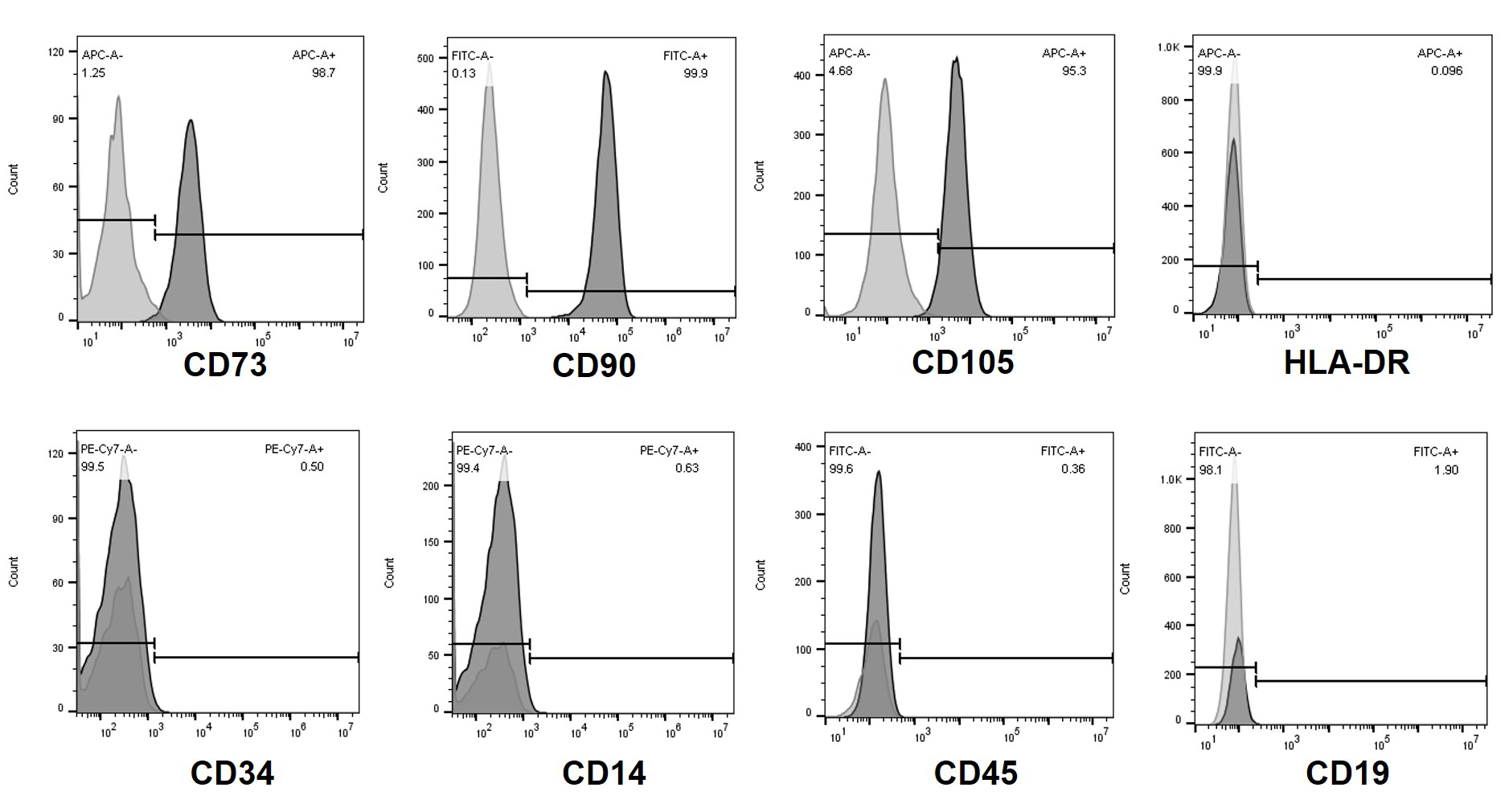

Supplement: Supplementary file 1 — Figure S1: The phenotype of the cells induced from iPSCs was examined by Flow Cytometry. Figure S2: Osteogenesis was restrained in iMSCs by the application of FKBP5 selective inhibitor SAFit2. Figure S3: iMSCs transplantation in the rat model of critical‐sized calvarial defect. Table S1: Antibodies used for cytometry flow. Table S2: Three shRNA sequences for FKBP5 knockdown were desired. Table S3: Primers used for real‐time qPCR. Table S4: Antibodies used for Westen Blot. Table S5: Antibodies used for co‐IP. [file JCMM-29-e70849-s001.zip › JCMM_70849_FigureS1.tif]

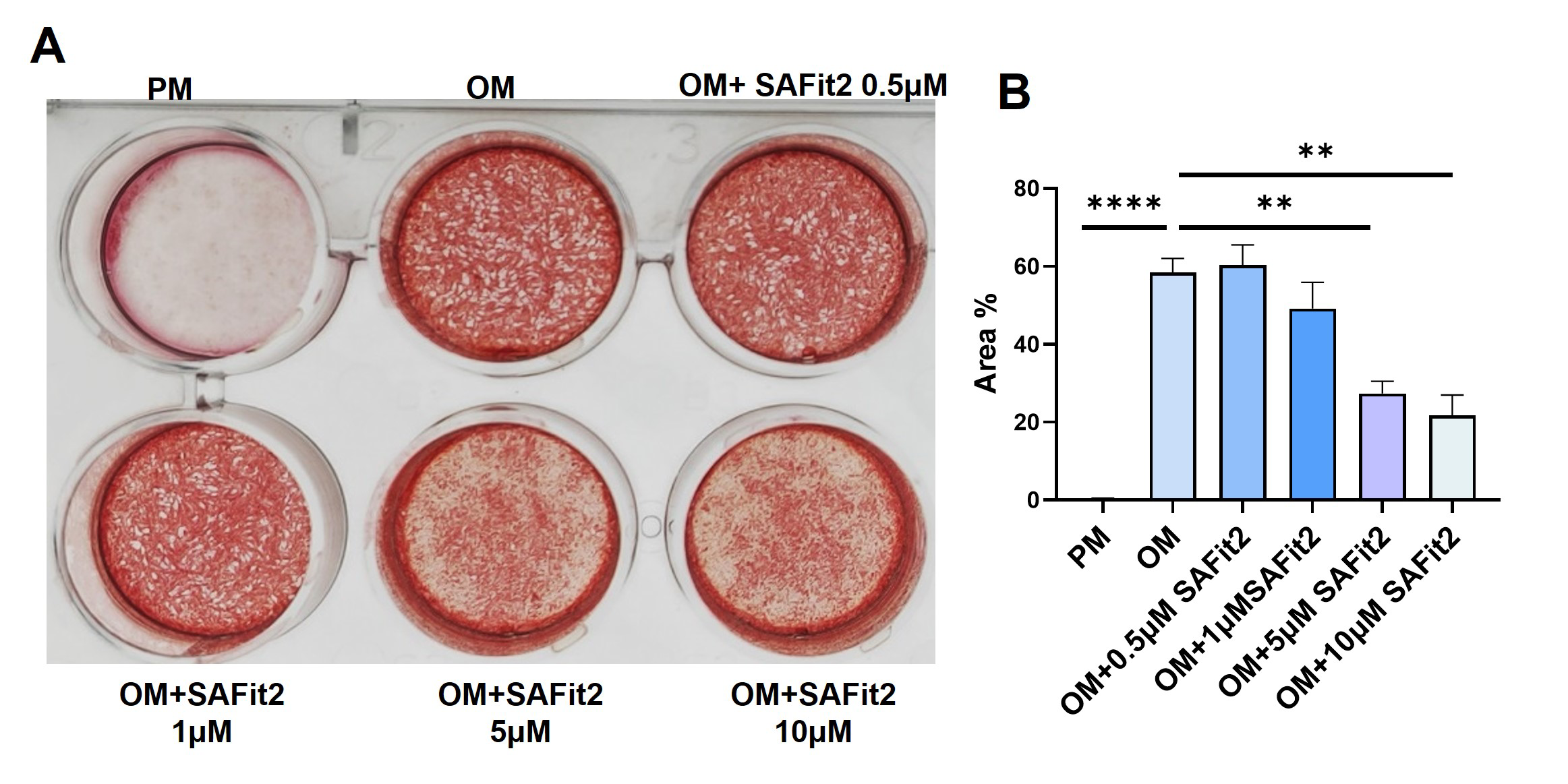

Supplement: Supplementary file 1 — Figure S1: The phenotype of the cells induced from iPSCs was examined by Flow Cytometry. Figure S2: Osteogenesis was restrained in iMSCs by the application of FKBP5 selective inhibitor SAFit2. Figure S3: iMSCs transplantation in the rat model of critical‐sized calvarial defect. Table S1: Antibodies used for cytometry flow. Table S2: Three shRNA sequences for FKBP5 knockdown were desired. Table S3: Primers used for real‐time qPCR. Table S4: Antibodies used for Westen Blot. Table S5: Antibodies used for co‐IP. [file JCMM-29-e70849-s001.zip › JCMM_70849_FigureS2.tif]

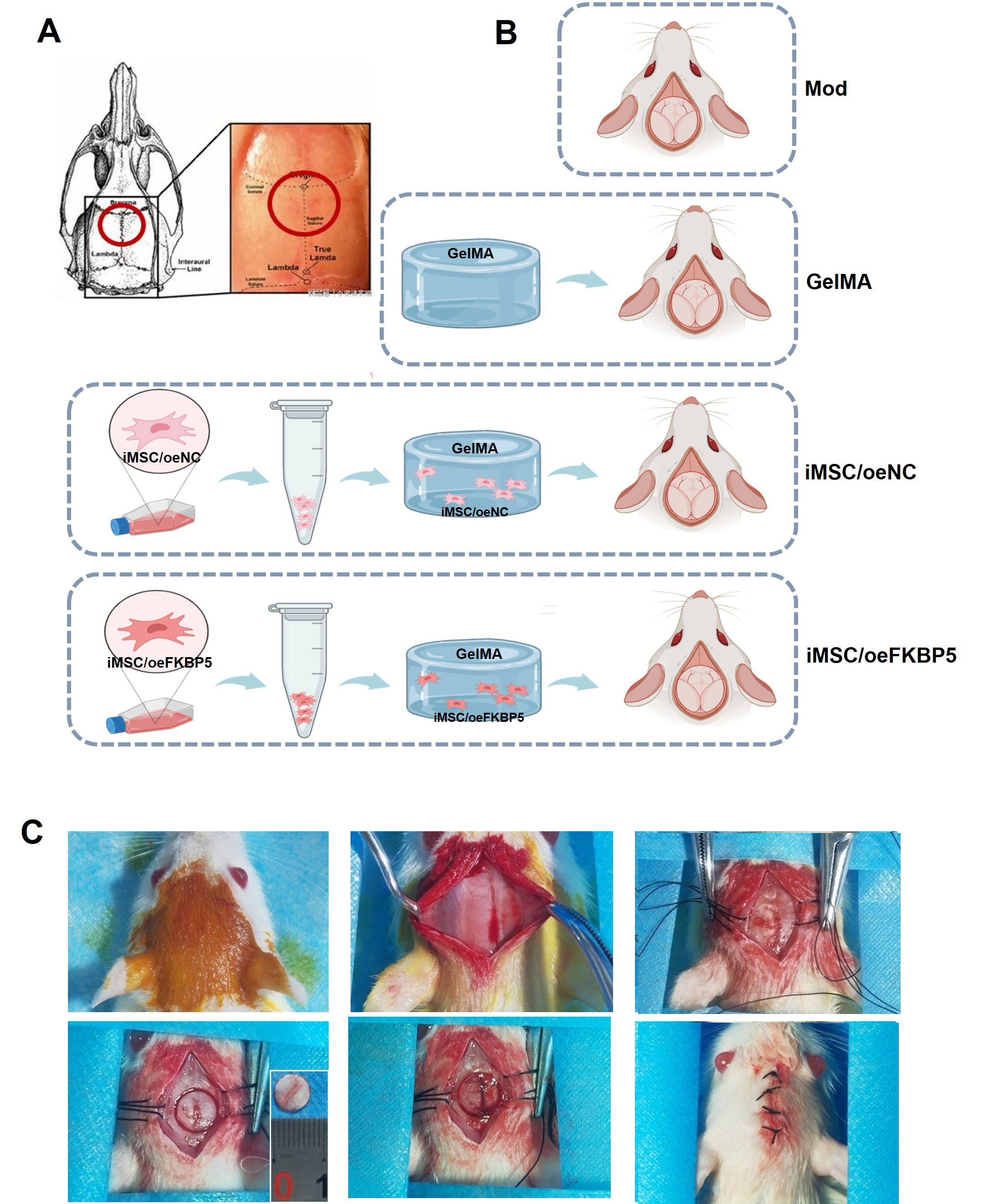

Supplement: Supplementary file 1 — Figure S1: The phenotype of the cells induced from iPSCs was examined by Flow Cytometry. Figure S2: Osteogenesis was restrained in iMSCs by the application of FKBP5 selective inhibitor SAFit2. Figure S3: iMSCs transplantation in the rat model of critical‐sized calvarial defect. Table S1: Antibodies used for cytometry flow. Table S2: Three shRNA sequences for FKBP5 knockdown were desired. Table S3: Primers used for real‐time qPCR. Table S4: Antibodies used for Westen Blot. Table S5: Antibodies used for co‐IP. [file JCMM-29-e70849-s001.zip › JCMM_70849_FigureS3.tif]
